# Children’s Filial Piety Changes Life Satisfaction of the Left-Behind Elderly in Rural Areas in China?

**DOI:** 10.3390/ijerph19084658

**Published:** 2022-04-12

**Authors:** Yaling Luo, Xiling Wu, Liao Liao, Hongmei Zou, Lulu Zhang

**Affiliations:** 1 School of Public Administration, Sichuan University, Chengdu 610065, China; luoyy@scu.edu.cn; 2 School of Politics and Public Administration, South China Normal University, Guangzhou 510006, China; l_liao@hotmail.com; 3 Xinyu Human Resources and Social Security Bureau of Jiangxi Province, Nanchang 330000, China; zouhongmei123@outlook.com; 4 College of Foreign Languages and Cultures, Sichuan University, Chengdu 610065, China; zhanglulu@scu.edu.cn

**Keywords:** filial piety, elderly, life satisfaction, rural areas, China

## Abstract

Along with the aging of the population and miniaturization of family structure, the problem of the left-behind elderly has become more and more prominent in China. According to the Report on the family development in China (2015) released by the National Health Commission of the People’s Republic of China, left-behind elderly people account for half of the total number of the elderly, of whom 10% live alone. The left-behind elderly not only suffer physiological obstacles such as body function decline, but also face a lack of support at the material level and loneliness at the spiritual level, which greatly affects their quality of life, accounting for their lower life satisfaction than that of the ordinary elderly. The rural areas of Sichuan Province are relatively backwards in terms of economic level and have limited pension security. Therefore, the left-behind elderly in rural areas are confronted with more complicated and severe pension problems compared with those in urban areas. Meanwhile, limited by economic and regional factors, a large number of rural labor forces in Sichuan Province have transferred to cities. These long-time migrant workers cannot provide material, spiritual and life care support for their left-behind parents in rural areas in a timely fashion, which changes their filial piety behaviors, and this affects the life satisfaction of the rural left-behind elderly. Therefore, it is necessary to understand the living conditions of empty-nest elderly and their children’s filial piety in rural areas of Sichuan province in order to verify the influence mechanism of filial piety on the life satisfaction of the elderly, and to explore how to improve the rural empty-nest elderly’s life satisfaction, enabling the elderly to live a healthy and happy life.

## 1. Introduction and Literature Review

### 1.1. Introduction

With the advancement of urbanization and the increase in the rural migrant population, the problem of left-behind elderly people in rural areas has become increasingly prominent. According to the statistics of the civil affairs department, there are more than 50 million left-behind elderly people in China’s rural areas. The left-behind elderly not only experience physical obstacles such as decline in physical function, they also face a lack of support at the material level and loneliness at the spiritual level [1], greatly affecting the quality of life of these people [2]. The life satisfaction of the left-behind elderly is lower than that of the general elderly. Compared with urban left-behind elderly, the rural left-behind elderly face more complex and severe elderly-care problems. As an area with a larger aging population, the problem of old-age care in rural areas is more acute and urgent. Sichuan Province is located in the economically underdeveloped southwest region of China. The social and economic conditions of its rural areas are relatively backward, and a large number of migrant workers leave to work. Due to the limitations of time and space, it is difficult for children to provide sufficient filial support. The left-behind elderly in rural areas face various problems such as lack of material support, spiritual loneliness [3], and lack of life care [4] The quality of life is adversely affected, with low levels of life satisfaction. Therefore, strengthening the research on the mechanisms affecting the influence of children’s filial piety on the life satisfaction of the elderly has practical significance for improving the life satisfaction of the rural left-behind elderly.

### 1.2. Literature Review

Filial piety refers to giving full regard to parents and obeying their will. Filial piety is a traditional Chinese virtue, and is deeply rooted in Chinese traditional culture. Filial piety takes the form of children’s respect for and obedience to, as well as their material and spiritual support for, their parents [5]. The degree of children’s filial piety determines the intergenerational relationship and the basic mode of supporting parents, which plays an important role in the elderly’s life satisfaction. Life satisfaction reflects the subjective quality of life of elderly individuals, which is an important indicator for measuring the level of a nation’s social pension system and the degree of social civilization [6]. Children play an important role in caring for elderly parents; in particular, adult children are regarded as an important source of emotional, physical and financial support for elderly parents [7]. At present, studies on the relationship between children and the elderly’s life satisfaction are mainly based on two aspects.

#### 1.2.1. The Relationship between the Objective Characteristics of Children and Life Satisfaction of the Elderly

Studies on the relationship between the objective characteristics of children and life satisfaction of the elderly mainly includes the influence of objective factors such as the number of children, sex, and living distance on the life satisfaction of the elderly [8]. Li, W. studies the influence of children’s factors on the life satisfaction of the urban elderly living alone [9]. The results show that elderly with more children have higher life satisfaction, but the order and gender of children have different effects on life satisfaction. Shi, Z. points out that in the early stage of old age, the improvement of daughters’ education levels is more effective in improving the elderly’s quality of life, and the educational level of sons plays a more significant role in the later stage [10]. Lian, Li and Huang study the effects of migrant workers on self-rated health and life satisfaction of their stay-at-home parents. They argue that migrant workers spend less time with their parents, which has a negative effect on parents’ health and life satisfaction [11].

#### 1.2.2. The Effect of Children Support on the Elderly’s Subjective Life Perception

Children support has great influence on the elderly’s life. Chen and Fang study the effects of children support on the subjective well-being of the elderly [12]. They argue that after receiving more comprehensive social support, the elderly’s overall evaluation of the quality of life will be improved accordingly. Through empirical analysis on the impact of intergenerational support on the elderly life satisfaction, Yu finds that there are urban–rural differences in life satisfaction of the elderly, and that the urban elderly have higher satisfaction than those in rural areas [13]. Additionally, intergenerational support has different impacts on urban and rural elderly life satisfaction [14]. In terms of offspring support, rural intergenerational support has a more significant impact on improving elderly life satisfaction. In terms of parental support, the rural elderly have more advantages in terms of time resources, while the urban elderly have more advantages with respect to economic resources [15].

In general, there are few studies on the relationship between filial piety of children and life satisfaction of the left-behind elderly in rural areas, and there are fewer empirical studies based on micro-survey data [16], with many research conclusions remaining controversial and questionable. In the context of the large gap between urban and rural economic development and the advanced stage of aging, it is beneficial to more comprehensively improving the life satisfaction of the left-behind elderly in rural areas to focus on their children’s filial piety. On the basis of describing the present situation of filial piety expectations, filial piety income, and differences in filial piety (filial piety includes three aspects: filial piety expectation, filial piety income, and differences in filial piety, and is measured using the filial piety scale, which includes six dimensions: children care, respect, greeting, pleasure, obedience and financial support. Filial piety expectation refers to the elderly’s subjective expectation of filial piety, filial piety income is the support obtained by the elderly in reality due to filial piety, and differences in filial piety is a measure of the degree to which filial piety expectation is met in reality, measured by subtracting the filial piety expectation score from the filial piety income score) among the rural empty-nest elderly, this study aims to verify the relationship between children’s filial piety and the life satisfaction of the rural left-behind elderly.

Therefore, this paper takes the rural left-behind elderly in Sichuan Province as the research object, studies the current situation of children’s filial piety and the life satisfaction of the rural left-behind elderly, and explores the effect of children’s filial piety on the life satisfaction of the rural left-behind elderly. Firstly, a large amount of data collection and literature review was carried out to clarify the background and significance of this research. By summarizing and commenting on relevant studies at home and abroad, the theoretical basis and research assumptions of this study are determined. Secondly, through field research and data analysis in rural areas of Sichuan Province, this paper discusses the current situation of the filial piety of children of left-behind elderly people and the status quo of life satisfaction of the elderly in rural areas of Sichuan Province. A logistic model was established to further study the relationship and gender differences between children’s filial piety and the life satisfaction of the rural left-behind elderly. Finally, according to the influence mechanism of filial piety expectation, filial piety support and differences in filial piety on the life satisfaction of the rural left-behind elderly, the key factors affecting the life satisfaction of the rural left-behind elderly are identified.

## 2. Theoretical Framework and Research Hypothesis

### 2.1. Relationship between Filial Piety of Children and Life Satisfaction of the Left-Behind Elderly in Rural Areas

It is generally believed that filial piety of children is a positive factor of life satisfaction of the elderly [17]. The more filial piety is offered, the higher the life satisfaction of the elderly is. However, filial piety is not only related to children’s objective support to the elderly, it is also closely related to the elderly’s subjective perceptions [18]. Filial piety should be a multi-dimensional concept that includes filial piety expectation, filial piety income, and differences in filial piety. The influence of filial piety expectation, filial piety income and differences in filial piety have an impact on the rural left-behind elderly’s life satisfaction. With the acceleration of urbanization, traditional living style and care arrangement have changed, and so has the form of intergenerational support [19]. Thus, we can draw research hypothesis 1 as follows:

**Research** **Hypothesis** **1** **(H1).** *Filial piety of children will promote life satisfaction of empty-nest elderly in rural areas.*

**Research** **Hypothesis** **1a** **(H1a).** *The higher filial piety expectation is, the lower life satisfaction of empty-nest elderly in rural areas will be.*

**Research** **Hypothesis** **1b** **(H1b).** *The higher filial piety income is, the higher life satisfaction of empty-nest elderly in rural areas will be.*

**Research** **Hypothesis** **1c** **(H1c).** *The greater differences in filial piety are, the higher life satisfaction of empty-nest elderly in rural areas will be.*

### 2.2. Gender Differences in the Influence of Children’s Filial Piety on Life Satisfaction of the Left-Behind Elderly in Rural Areas

From the perspective of life course, the weakness of elderly women is largely caused by history [20]. In many life stages before old age, women face injustice in many respects, such as in terms of education, employment, political participation, and so on. Years of accumulated disadvantage have led to elderly women’s low economic security and high dependency on their children. Therefore, the filial piety of children will have a more significant impact on elderly women’s life satisfaction [21]. At the same time, according to Maslow’s hierarchy of needs, the lack of economic independence leads to stronger basic survival and security needs, so elderly women are eager for more life care and economic support from their children. When male elderly have more abundant social capital and higher personal income that can basically meet their low-level needs like survival, they will further pursue the satisfaction of high-level needs such as social interaction, respect and self-realization, etc. [22]. They yearn for respect, obedience and caring greetings from their children. Therefore, we draw the following hypotheses:

**Research** **Hypothesis** **2** **(H2).** *Compared with male left-behind elderly in rural areas, children’s care and economic support will have a more significant impact on life satisfaction of female left-behind elderly.*

**Research** **Hypothesis** **3** **(H3).** *Compared with female left-behind elderly in rural areas, children’s respect, greetings, pleasure and obedience will have a significant impact on life satisfaction of male left-behind elderly.*

## 3. Data and Methods

### 3.1. Data Sources

The data for this study were collected from rural empty-nest elderly in Sichuan Province, which was carried out as part of the project of *Research on New Dilemma of Rural Empty-nest elderly Pension Security in Sichuan Province*. The research was conducted from August 2016 to May 2017. The research objects mainly consisted of the questionnaire data of the elderly aged 60 and above (the full text of the questionnaire is shown in the Appendix A), and family questionnaire data including basic information of the elderly, their children, etc. The total sample size of the empty-nest elderly was 1016.

In terms of sampling, the smallest unit of sampling was a town. First of all, we took GDP as the measure of economic development level. According to the GDP level of Sichuan Province, Chengdu, Leshan and Suining were selected, which respectively ranked first, eighth, and fifteenth in the GDP ranking for Sichuan province in 2016, representing the best, average, and poor cities in terms of economic development. Afterwards, Qionglai, Jingyan and Pengxi were selected from subordinate areas of Chengdu, Leshan and Suining by random sampling. Finally, 3 towns in Qionglai, Jingyan and Pengxi were randomly selected as research sites. Because of short distances (it is not far between the towns), population mobility (going to a fair and visiting one’s relatives or friends), and other reasons, the final survey results included more than three towns at each site. Three towns were selected from Qionglai City in Chengdu, and the final survey sample included 32 villages in 4 towns; 3 towns were selected from Jingyan County in Leshan, and the final survey sample included 41 villages in 6 towns; 3 towns were selected from Pengxi County in Suining, and the final survey sample included 141 villages in 13 towns.

A total of 1044 questionnaires were distributed. After processing the data and removing missing values, 1016 of them were valid, i.e., the questionnaire recovery rate was 97.32%.

The authors sorted the data collected by the survey and entered it into SPSS 22.0, and analyzed the reliability and validity of the data. The Alpha coefficient of the reliability statistics of the specific results is greater than 0.9, indicating that the reliability of the scale in this paper is good. At the same time, the KMO and Bartlett sphericity test for factor analysis were used, and the resulting KMO statistic was close to 1 and significant, so the survey data had statistical significance. Therefore, the measured data could be used in this research to analyze the influence of children’s filial piety on the life satisfaction of the elderly.

### 3.2. Variable Measurement

Dependent variable. The dependent variable in this paper is life satisfaction of the left-behind elderly in rural areas of Sichuan Province, measured by the question “I am satisfied with my life”. Participants should answer “fully agree” (5 points), “agree” (4 points), “generally agree (3 points), “disagree” (2 points), or “totally disagree” (1 point) to measure their life satisfaction. The higher the score is, the higher their degree of felt life satisfaction.

Explanatory variable. The explanatory variable is children’s filial piety, which is reflected by filial piety expectation, filial piety income and differences in filial piety. We use the filial piety scale to measure the degree of children’s filial piety. The filial piety scale includes six dimensions, namely children care, respect, greeting, pleasure, obedience and financial support.

Filial piety expectation. We measure the filial piety expectation of the elderly by asking them six questions: how much they expect to be cared for, respected, greeted, pleased, obeyed and financially supported by their children. The degree of expectation is determined on the basis of the answers “not at all” (1 point), “very little” (2 points), “about average” (3 points), “not a little” (4 points) or “very much” (5 points). The higher the score is, the greater the expectation is. Filial piety expectation is divided into three levels: low expectation (6 to 18 points), general expectation (19 to 24 points), and high expectation (25 to 30 points).

Filial piety income. Contrary to filial piety expectation, filial piety income is measured by asking the elderly whether their children actually provide them with care, respect, greeting, pleasure, obedience and financial support. Likewise, the elderly determine the degree of his children’s filial piety in practice by answering “not at all” (1 point), “very little” (2 points), “about average” (3 points), “not a little” (4 points) or “very much” (5 points), with higher scores indicating higher filial piety income. Filial piety income is divided into three levels: low income (6 to 20 points), middle income (21 to 24 points), and high income (25 to 30 points).

Differences in filial piety. Differences in filial piety is an integral measure of the degree of children’s filial piety, and takes both filial piety expectation and filial piety income into account. Differences in filial piety is measured by subtracting the filial piety expectation score from the filial piety income score. If the score is positive, it means that the elderly’s filial piety income exceeds their filial piety expectations of their children, and vice versa. This paper divides filial piety expectation and filial piety income into four types: high expectation–high income, high expectation–low income, low expectation–high income, and low expectation–low income. Six dimensions of care difference, respect difference, pleasure difference, greeting difference, obedience difference and financial support difference are also contained in differences in filial piety. Consistent with overall differences in filial piety, the score of each sub-dimension is the score of filial piety income minus the score of filial piety expectation in each corresponding dimension.

Controlled variables. The controlled variables in this study mainly include region, gender, marital status, number of children, education level, annual income and expenditure and self-rated health of the empty-nest elderly in rural areas.

## 4. Analytical Results

### 4.1. Sample Description

Table 1 contains the descriptive statistics and gender differences for the main variables in this study. It is shown in the table that the life satisfaction of the rural left-behind elderly is generally higher, and that the level is higher in males than in females. In terms of personal annual income and expenditure, the overall level of men is higher than that of women, indicating that women are economically disadvantaged. In regard to education level, the overall education level of the rural left-behind elderly is low, and male education level is higher than female as a whole. With respect to marital status, female left-behind elderly have a higher rate of losing their spouse because they have a longer life expectancy than men. Considering self-rated health, elderly women’s physical condition is better men’s. With respect to children’s filial piety, female filial piety expectation is higher than male, while filial piety income is lower, and the overall differences in filial piety of females is higher than that of males.

### 4.2. Children’s Filial Piety with Respect to Left-Behind Elderly in Rural Areas

Overall filial piety. As shown in Table 2, the average values of filial piety expectation and filial piety income are 21.40 and 16.18, respectively. The overall average of differences in filial piety is −5.21, since the overall score of filial piety income was lower than that of filial piety expectation. Differences in filial piety are negative in general, indicating that the degree of children’s filial piety is generally not up to the expectation of the left-behind elderly in rural areas. In addition, there are gender differences in the perception of filial piety of the left-behind elderly in rural areas. Male left-behind elderly’s filial piety expectations are lower than those of female left-behind elderly, but their filial piety income is higher than that of females, and their differences in filial piety are also smaller. Since male left-behind elderly have a dominant position in terms of social capital, their economic and emotional independence is higher than that of women, so their filial piety expectations will be relatively low. Rural male left-behind elderly’s family status is higher than that of females, and children will give them more support. In short, children show more filial piety to their father than to their mother.

Dimensions of filial piety and filial piety levels. Overall situation of each dimension. First of all, in each dimension of filial piety expectation, the rural left-behind elderly have high expectations with respect to greeting, care and respect from their children. Male left-behind elderly have lower expectations of greeting, care, pleasure, obedience and financial support than females, except for the expectation of respect, which indicates that male elderly have a greater need for higher levels of respect. Secondly, in all dimensions of filial piety income, the rural left-behind elderly have the highest respect income, while care income and financial income are the lowest, and the male left-behind elderly have higher filial piety income than women in all six aspects. It is very difficult for migrant workers to provide their parents with timely care. Meanwhile, limited by their own financial income and living pressure, migrant workers’ financial support ability is low, so care income and financial income of the elderly are low. While children live far away from home, there is a lack of daily communication between children and parents, which helps avoid intergenerational contradiction to a certain extent, so the elderly’s respect income is higher. Finally, in each dimension of differences in filial piety, the rural left-behind elderly have the highest absolute value of care difference, financial difference and greeting difference, and female have higher absolute value than male, illustrating that children’s actual support are far from the elderly’s expectations in three aspects of daily care, financial support and greeting care.

Filial piety levels. A total of 24.04% of the rural left-behind elderly hold high expectations for their children’s filial piety behavior. However, only 2.07% of the elderly have high filial piety income, and 85.32% have lower filial piety income. At the same time, 53.6% of elderly people belong to the “low expectation–low income” and “high expectation–low income” groups. This reveals that in rural areas of Sichuan, the degree of children’s filial piety is generally low, and hasn’t reached the elderly’s expectation.

### 4.3. Life Satisfaction of the Rural Left-Behind Elderly

**Figure 1:**
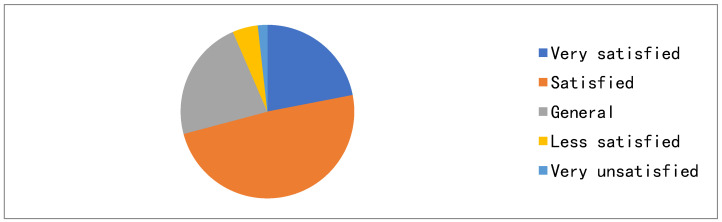
Current life satisfaction of empty-nest elderly in rural areas.

In this survey, the average life satisfaction score of the rural left-behind elderly was 3.85 points, and male life satisfaction was slightly higher than that of females. As shown in Figure 1, the proportion of the left-behind elderly who were “very satisfied” and “satisfied” with their overall situation of life was as high as 70.87%. It can be seen that rural empty-nest elderly people in Southwest China have high overall life satisfaction. This is contrary to the view that the existing rural left-behind elderly have a low quality of subjective life satisfaction. Life satisfaction is a relative concept that not only contains a description of the current living conditions, but also a comparison with previous living ones. Although the elderly in rural areas live a life of homespun cotton and vegetables, they still feel contented compared with their previous life.

#### 4.3.1. Relationship between Children’s Filial Piety and Life Satisfaction of the Rural Left-Behind Elderly

Relationship between filial piety expectation, filial piety income and life satisfaction. Filial piety expectation and life satisfaction. From the perspective of subjective will, filial piety expectation depicts the expectations of the elderly for their children’s filial piety. When analyzing the impact of children’s filial piety on the life satisfaction of the rural left-behind elderly, the method of gradually increasing the influencing factors was used to perform multiple fitting regressions. Firstly, Model 1 is the benchmark model, and only the regional variable and the gender variable of the left-behind elderly were introduced into Model 1 to examine the correlation between the explanatory variable and the explained variable. Secondly, on the basis of Model 1, the variables of personal marital status and the number of children are introduced. Thirdly, the variables of personal education level and income were introduced on the basis of Model 2. Finally, the variables of personal self-assessed health were introduced on the basis of Model 3. In this way, the scientificity and reliability of the regression results were continuously improved. From Model 1 to Model 3 in Table 3, filial piety expectation is significantly negatively correlated with the life satisfaction of the elderly. The more the elderly expect their children to provide care, respect, greetings, pleasure, obedience and financial support, the lower their life satisfaction is. This is because those elderly whose filial piety expectation is high have higher material, spiritual and financial dependence on their children. Their poor physical condition or negative subjective attitude leads to low independence of personal life, which will reduce their viability and lead to low life satisfaction.

In terms of gender differences, filial piety expectation does not have a significant impact on females, but has a significantly negative effect on the male elderly. Comparing the R^2^ of Model 5 and Model 6, it can be seen that the model is better explained for the female elderly than for the male.

In terms of filial piety expectation levels (as shown in Table 4), “medium expectation” increases life satisfaction.

Filial piety income and life satisfaction. Filial piety income is objectively received filial piety support for the elderly. From Model 1 to Model 4 in Table 3, we gradually add various variables, and filial piety income is always positively correlated with the life satisfaction of the elderly. The elderly have a higher life satisfaction when they receive more financial support, spiritual comfort, and life care from their children indeed.

**Figure 2:**
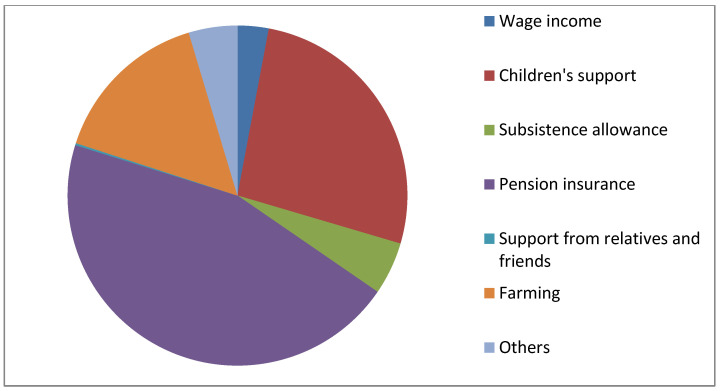
Main sources of financial income.

In terms of filial piety income level (as shown in Table 4), with low filial piety income as the reference group, medium filial piety income and high filial piety income have significant effects on life satisfaction of the elderly in Model 1 and Model 2. In Model 3 and Model 4, filial piety income does not have a significant impact on their life satisfaction, which may be explained by personal income and expenditure. Personal annual income includes children’s financial support, which is one of main sources of income for the elderly. The main sources of income of the respondents in this study are shown in Figure 2.

Other variables and life satisfaction. As shown in Table 3, region, gender, marriage, number of sons, annual income, annual expenditure, self-rated health, filial piety expectation, and filial piety income all have different degrees of impact on the life satisfaction of the elderly. Among them, regional economic level is negatively correlated with life satisfaction, that is, the higher the level of regional economic development, the lower the life satisfaction of the elderly. This is because in areas with high economic development, the relative weakness of empty-nest elderly becomes more obvious, and their quality of life is significantly lower than that of non-empty-nest elderly. On the contrary, marital status (with or without a spouse), number of sons, personal income and expenditure, and physical condition are all positively related to life satisfaction of the elderly. That is, the elderly will have higher life satisfaction if they have a spouse, better physical condition, more number of sons or higher personal income and expenditure. Among them, the number of sons has a significant impact on life satisfaction, while the number of daughters is not. In rural areas, it still exists the concept that raising sons helps carry on the family line, and the elderly with more sons can gain more sense of security.

In addition, there are gender differences in the factors affecting life satisfaction of empty-nest elderly in rural areas. Comparing Model 5 with Model 6, it can be seen that marital status has a significantly positive impact on female quality of life, but that this is not significant for men. Region and annual income have a significant impact on male empty-nest elderly; in particular, income significantly improves their life satisfaction, while women are not significantly affected by these two factors.

**Differences in filial piety and life satisfaction.** Differences in filial piety describes the extent to which the rural left-behind elderly are satisfied in terms of care, respect, greeting, pleasure, obedience, and financial support. Table 5 describes the relationship between differences in filial piety and life satisfaction of the elderly. This paper repeats the logical model with respect to filial piety expectation and filial piety income, in order to understand the mechanism of how differences in filial piety affect the life satisfaction of rural empty-nest elderly through comparative analysis.

From Model 1 to Model 4, even if various variables are added gradually, differences in filial piety always have a significant influence on life satisfaction of the elderly at the 0.001 level. The greater the difference in filial piety, the higher the life satisfaction.

In terms of different types of difference in filial piety, different types of filial differences have different effects on life satisfaction of the rural left-behind elderly (as shown in Table 6). With “low expectation–low income” as the reference group, “low expectation–high income” type and “high expectation–high income” type of differences in filial piety will significantly improve life satisfaction of the empty-nest elderly, but “high expectation–low income” types of difference in filial piety will not have a significant impact on life satisfaction. This indicates that children’s actual filial piety support behavior is a key factor affecting the elderly’s happiness. In addition, there is a gender differences in the impact of differences in filial piety on life satisfaction of rural left-behind elderly. “High expectation–high income” types of differences in filial piety will significantly improve females’ life satisfaction, while “low expectation–high income” types of differences in filial piety will significantly enhance male subjective life satisfaction.

#### 4.3.2. Relationship between Each Dimension of Filial Piety and the Life Satisfaction of the Rural Left-Behind Elderly

Table 7 describes the impact of six specific dimensions of filial piety on the life satisfaction of the rural left-behind elderly: care, respect, greeting, pleasure, obedience, and financial support. We analyze the factors that have a substantial impact on life satisfaction of the rural left-behind elderly in terms of filial piety expectation, filial piety income and differences in filial pietys.

Dimensions of filial piety expectation and life satisfaction of left-behind elderly in rural areas. Among all inner dimensions of filial piety expectation, according to Model 1 to Model 4, obedience, financial support and respect of filial piety expectation have a significant impact on the life satisfaction of the rural left-behind elderly. Expectation of obedience and financial expectations have a negative effect on life satisfaction, while expecation of respect has a positive effect. Because the elderly who have high expectation of obedience and financial expectations do not obtain their children’s compliance in real life, their financial independence is low, resulting in low satisfaction. Those people who have high esteem need may have met lower-level needs of survival and safety, and thus have high life satisfaction.

In addition, there is a gender difference in the mechanism of how the internal factors of filial piety expectation affect the life satisfaction of empty-nest elderly. Female filial piety expectation is mainly concentrated in financial expectations, while the male elderly mainly focus on expectation of respect. Because of female disadvantages accumulated in previous life stages, women’s financial security is more vulnerable during old age, and the female elderly’s financial security is significantly lower than that of males [23], so women’s financial expectations are higher than men’s. With rich social capital and high personal income, men are eager for higher-level need of respect, which has a greater impact on their life.

Dimensions of filial piety income and life satisfaction of left-behind elderly in rural areas. In terms of the internal dimensions of filial piety income, from Model 1 to Model 4, respect factor and financial factor significantly affect the life satisfaction of the elderly. The more respect and financial support the elderly receive from their children, the higher their subjective quality of life is. Furthermore, financial support has always been the core impact factor of life satisfaction for rural empty-nest elderly.

Considering gender differences, income of respect received from children significantly improves women’s life satisfaction, while income of financial support significantly enhances men’s life satisfaction. The status of women is still slightly lower than that of men in rural areas, and they are more vulnerable to discrimination in work and life, so the respect of children can significantly improve their sense of satisfaction. Correspondingly, men’s social capital is more advantageous, daily activities are more abundant, and they can take pleasure and comfort from their relatives and friends. Additionally, men’s life expectancy is shorter than women’s, and they have a lower possibility of losing their spouse, so they can usually be taken care by their spouse. Therefore, men have less spiritual dependence and life dependence on their children, but because of the loss of working ability and low personal income, financial income received from their children will significantly affect their life satisfaction.

Dimensions of differences in filial piety and life satisfaction of left-behind elderly in rural areas. In regard to internal dimensions of differences in filial piety, from Model 1 to Model 3, it is shown that obedience and financial difference are the core factors affecting life satisfaction of the rural left-behind elderly. The elderly have higher life satisfaction when children are more willing to follow obey will and more financial support is given to the elderly than expected.

With respect to gender differences, there is a single dimension of differences in filial piety affecting female empty nesters’ life satisfaction. Women are only affected by financial difference, while the male empty-nest elderly are affected by multiple differences. Greeting difference, obedience difference and financial difference will significantly improve male empty nester’s life satisfaction. This is because the financial security of male elderly people is generally greater than that of females. Women’s individual financial need is stronger, while men’s needs are more complicated. Men’s quality of life is not only affected by their children’s financial support, but their children’s spiritual comfort and obedience also have an impact.

## 5. Conclusions and Discussion

### 5.1. Conclusions

This study used the investigation of the current situation of empty nesters in Sichuan rural areas through the project of Research on New Dilemma of Rural Empty-nest elderly Pension Security in Sichuan Province, and explored the mechanisms and gender differences at play in how children’s filial piety affects the life satisfaction of the elderly. The results show that our research hypotheses have all been confirmed. First, filial piety expectation has a negative impact on the life satisfaction of the elderly. The higher the filial piety the empty-nest elderly in rural areas expect, the lower the life satisfaction they have. Second, filial piety income and differences in filial piety significantly improve life satisfaction among the elderly. The more filial piety support behaviors children indeed show and the more filial piety income exceeds filial piety expectation, the higher the life satisfaction experienced by the elderly. Third, there are gender differences in the influence of filial piety on the life satisfaction of empty nesters. Compared with male rural left-behind elderly, children’s care and financial support have a more significant impact on the life satisfaction of female rural left-behind elderly. However, compared with female rural left-behind elderly, children’s respect, greetings, pleasure and obedience have a more significant impact on male left-behind elderly’s life satisfaction. Specifically, female empty nesters’ filial piety expectations are concentrated in financial expectation. The higher the financial expectation, the lower the life satisfaction. Meanwhile, the filial piety expectations of male empty nesters are concentrated in the expectation of respect. The higher the expectation of respect, the higher the life satisfaction. Among all dimensions of filial piety income, women have higher life satisfaction when they receive higher income of respect, and the male elderly’s financial income has a significantly positive impact on their life satisfaction. In regard to differences in filial piety, female empty nesters’ differences in filial piety has a single impact on life satisfaction. Only financial difference has a significant effect on female life satisfaction, while male empty-nest elderly have diversified characteristics, of which greeting difference, obedience difference, and financial difference are positively correlated with life satisfaction. In general, in these six specific dimensions of filial piety, namely care, respect, greeting, pleasure, obedience and financial support, the financial dimension is always the key factor affecting filial piety perception among the elderly, and financial expectation, financial income and financial difference all have a significant impact on the life satisfaction of the elderly. Conversely, pleasure expectation, pleasure income, and pleasure difference have no significant effect.

### 5.2. Discussion

This study has important theoretical and practical significance. On the one hand, this study starts from the filial piety of the children of the rural left-behind elderly in Sichuan Province and the status quo of life satisfaction of the elderly, and uses the filial piety scale to explore the effect of filial piety expectations, filial piety support and differences in filial pietys on the life satisfaction of the rural left-behind elderly. The mechanism of influence of children’s caring, respect, greetings, pleasure, obedience and financial support towards the elderly over the life satisfaction of the elderly was investigated, clarifying the key factors that have a substantial impact on the life satisfaction of the rural left-behind elderly with respect to children’s filial piety, and analyzing the gender differences in the factors affecting the life satisfaction of the rural left-behind elderly, which is helpful for enriching relevant theories of life satisfaction of the rural left-behind elderly in Sichuan Province and expanding the theoretical horizon of rural pension issues. On the other hand, analyzing the current situation of children’s filial piety among the left-behind elderly in rural Sichuan Province and exploring the influencing factors of children’s filial piety will contribute to guiding children to identify the actual pension needs of the elderly, and accurately promote the building of family pension support systems, increasing the elderly’s filial support and differences in filial piety, and improving children’s filial piety, thereby improving the life satisfaction of the elderly.

The contribution of this paper is to analyze the connotation of children’s filial piety from multiple angles, find the key factors affecting the life satisfaction of the rural left-behind elderly related to children’s filial piety, and compare and analyze the gender differences between children’s filial piety and the life satisfaction of the rural left-behind elderly, which enriches the previous research perspectives. In addition, the limitation of this study is that, on the one hand, the research depth needs to be expanded. The theoretical research regarding the relationship between children’s filial piety and the life satisfaction of the rural left-behind elderly is still in its infancy, and there are few theories and literature in this area. Therefore, this paper lacks sufficient theoretical support and literature research. On the other hand, comparative studies are insufficient. This paper focuses on the overall evaluation and analysis of regional differences in the life satisfaction of the rural left-behind elderly in Sichuan Province, and lacks a comparison of the difference in life satisfaction between the urban and rural left-behind elderly and the left-behind elderly and non-left-behind elderly. In addition, longitudinal dynamic comparative research on life satisfaction of rural left-behind elderly at different time points also needs to be strengthened, all of which need to be studied by subsequent scholars.

## Figures and Tables

**Table 1:** Descriptive statistics of main variables.

	Total (N = 1016)		Female (N = 365)		Male (N = 651)	
	Mean	SD	Mean	SD	Mean	SD
Life satisfaction	3.85	0.88	3.78	0.89	3.89	0.87
Age	71.63	7.10	71.18	7.07	71.88	7.12
Log (annual income)	8.42	1.05	8.30	1.04	8.49	1.05
Log (annual expenditure)	8.71	0.77	8.62	0.76	8.75	0.77
Education level (primary school = 1)	0.85	0.36	0.94	0.24	0.80	0.40
Marital status (with a spouse = 1)	0.71	0.46	0.64	0.48	0.74	0.44
Number of sons	1.31	0.91	1.37	0.95	1.27	0.88
Number of daughters	1.20	1.05	1.23	1.07	1.18	1.04
Self-rated health (score)	3.04	1.16	3.13	1.10	2.99	1.19
Filial piety expectation (score)	21.40	5.05	21.68	4.96	21.24	5.10
Filial piety income (score)	16.18	4.22	15.94	4.40	16.32	4.12
Differences in filial piety (score)	−5.21	5.79	−5.74	5.84	−4.9	5.75

Note. Variable order.

**Table 2:** Filial piety expectation, filial piety income and differences in filial piety among rural empty-nest elderly.

	Total Mean (SD)	Range	Female Mean (SD)	Male Mean (SD)	Coefficient/*p* Value
Overall filial piety expectation, mean (SD)	21.40 (5.05)	(6, 30)	21.68 (4.96)	21.24 (5.10)	3.87 ***
Care expectation	3.68 (1.23)	(1, 5)	3.81 (1.19)	3.62 (1.25)	−0.01
Expectation of respect	3.67 (0.95)	(1, 5)	3.66 (0.96)	3.68 (0.94)	0.13 *
Greeting expectation	3.72 (0.97)	(1, 5)	3.74 (0.98)	3.71 (0.97)	−0.04
Pleasure expectation	3.62 (0.98)	(1, 5)	3.66 (0.96)	3.61 (0.99)	0.07
Expectation of obedience	3.25 (1.08)	(1, 5)	3.29 (1.03)	3.23 (1.11)	−0.07
Financial expectation	3.44 (1.21)	(1, 5)	3.53 (1.16)	3.39 (1.23)	−0.09 **
Filial piety expectation levels, N (%)					
High expectation	244 (24.04)	(6, 18)	84 (23.01)	160 (40.00)	0.27
Medium expectation	557 (54.88)	(19, 24)	214 (58.63)	343 (52.77)	0.12
Low expectation	214 (21.08)	(25, 30)	67 (18.36)	147 (22.62)	Control group
Overall filial piety income, mean (SD)	16.18 (4.22)	(6, 30)	15.94 (4.39)	16.32 (4.12)	2.95 ***
Care income	2.32 (1.03)	(1, 5)	2.32 (1.02)	2.32 (1.03)	0.02
Respect income	3.02 (0.82)	(1, 5)	2.95 (0.82)	3.06 (0.81)	0.13 *
Greeting income	2.88 (0.91)	(1, 5)	2.84 (0.93)	2.91 (0.89)	0.06
Pleasure income	2.83 (0.85)	(1, 5)	2.77 (0.87)	2.86 (0.83)	−0.56
Obedience income	2.76 (0.81)	(1, 5)	2.73 (0.82)	2.78 (0.80)	0.02
Financial income	2.37 (1.01)	(1, 5)	2.33 (1.00)	2.39 (1.02)	0.16 ***
Filial piety income levels, N (%)					
High income	21 (2.07)	(6, 20)	10 (2.74)	11 (1.69)	0.38 *
Medium income	128 (12.61)	(21, 24)	37 (10.14)	91 (14.00)	0.23 **
Low income	866 (85.32)	(25, 30)	318 (87.12)	548 (84.31)	Control group
Overall differences in filial piety, mean (SD)	−5.21 (5.79)	(−24, 19)	−5.74 (5.84)	−4.91 (5.75)	4.01 ***
Care difference	−1.36 (1.56)	(−4, 4)	−1.48 (1.51)	−1.29 (1.58)	0.01
Respect difference	−0.65 (1.15)	(−4, 4)	−0.71 (1.16)	−0.62 (1.14)	−0.02
Greeting difference	−0.84 (1.19)	(−4, 4)	−0.90 (1.21)	−0.81 (1.18)	0.05
Pleasure difference	−0.80 (1.13)	(−4, 4)	−0.89 (1.17)	−0.74 (1.10)	−0.04
Obedience difference	−0.49 (1.06)	(−4, 3)	−0.56 (1.07)	−0.45 (1.05)	0.07 *
Financial difference	−1.07 (1.53)	(−4, 4)	−1.20 (1.49)	−1.0 (1.55)	0.12 ***
Differences in filial piety levels, N (%)					
High expectation–high income	297 (29.26)	(21, 30), (16, 30)	109 (29.86)	188 (28.92)	0.28 ***
High expectation–low income	279 (27.49)	(21, 30), (6, 16)	121 (33.15)	158 (24.31)	0.01
Low expectation–high income	174 (17.14)	(6, 21), (16, 30)	47 (12.88)	127 (19.54)	0.49 ***
Low expectation–low income	265 (26.11)	(6, 21), (6, 16)	88 (24.11)	177 (27.23)	Control group

* *p* < 0.05. ** *p* < 0.01. *** *p* < 0.0001.

**Table 3:** Relationship between filial piety expectation, filial piety income, and life satisfaction of empty-nest elderly in rural areas.

Variables	Model 1	Model 2	Model 3	Model 4	Model 5 (Female)	Model 6 (Male)
Region	−0.10 **	−0.11 **	−0.10 **	−0.11 **	−0.07	−0.12 **
Gender (male = 1)	0.11 *	0.11 *	0.09	0.07		
Marital status (with a spouse = 1)		0.14 *	0.14 *	0.12 *	0.21 *	0.07
Number of sons		0.10 **	0.09 **	0.11 ***	0.12 *	0.09 *
Number of daughters		0.02	0.01	0.02	0.06	−0.01
Education level (primary school = 1)			0.12	0.13	0.27	0.11
Log (annual income)			0.13 ***	0.08 **	0.07	0.09 *
Log (annual expenditure)			0.06	0.08 *	0.05	0.09
Physical condition				0.23 ***	0.23 ***	0.23 ***
Filial piety expectation	−0.01 **	−0.02 **	−0.01 *	−0.01	0.01	−0.02 *
Filial piety income	0.06 ***	0.06 ***	0.05 ***	0.04 ***	0.05 ***	0.04 ***
R^2^	8.53%	9.83%	12.92%	20.84%	25.17%	19.20%
df	4	7	10	11	10	10

* *p* < 0.05. ** *p* < 0.01. *** *p* < 0.001.

**Table 4:** Relationship between filial piety expectation level, filial piety income level, and life satisfaction.

Variables	Model 1	Model 2	Model 3	Model 4	Model 5 (Female)	Model 6 (Male)
Region	−0.05	−0.07 *	−0.06	−0.07 *	−0.03	−0.09 *
Gender (male = 1)	0.13 *	0.13 *	0.09	0.07		
Marital status (with a spouse = 1)		0.18 **	0.16 **	0.14 *	0.25 **	0.07
Number of sons		0.10 **	0.09 **	0.11 ***	0.14 **	0.08 *
Number of daughters		0.05	0.04	0.04	0.11 *	0.01
Education level (primary school = 1)			0.07	0.09	0.16	0.08
Log (annual income)			0.16 ***	0.11 ***	0.11 *	0.11 **
Log (annual expenditure)			0.07	0.10 **	0.07	0.11 *
Physical condition				0.24 ***	0.24 ***	0.24 ***
Low expectation (reference group)						
Medium expectation	0.12	0.12	0.14 *	0.12	0.30 *	0.04
High expectation	−0.12	−0.13	−0.08	−0.03	0.16	−0.12
Low income (reference group)						
Medium income	0.26 **	0.25 **	0.14	0.12	0.11	0.12
High income	0.47 *	0.46 *	0.28	0.21	0.30	0.12
R^2^	2.93%	4.65%	9.52%	17.98%	21.40%	17.01%
df	6	9	12	13	12	12

* *p* < 0.05. ** *p* < 0.01. *** *p* < 0.001.

**Table 5:** Relationship between differences in filial piety and life satisfaction of empty-nest elderly in rural areas.

Variables	Model 1	Model 2	Model 3	Model 4	Model 5 (Female)	Model 6 (Male)
Region	−0.07 *	−0.09 *	−0.07 *	−0.08 *	−0.04	−0.10 *
Gender (male = 1)	0.11	0.11	0.07	0.06 *		
Marital status (with a spouse = 1)		0.17 **	0.16 **	0.14 ***	0.24 *	0.08
Number of sons		0.11 **	0.10 **	0.11	0.14 **	0.09 *
Number of daughters		0.03	0.02	0.03	0.09 *	−0.01
Education level (primary school = 1)			0.10	0.12	0.17	0.11
Log (annual income)			0.14 ***	0.09 **	0.09	0.10 **
Log (annual expenditure)			0.07	0.09 *	0.07	0.10 *
Physical condition				0.24 ***	0.24 ***	0.24 ***
Differences in filial piety	0.03 ***	0.03 ***	0.03 ***	0.02 ***	0.02 *	0.02 ***
R^2^	4.95%	6.59%	10.48%	18.94%	20.57%	18.32%
df	3	6	9	10	9	9

* *p* < 0.05. ** *p* < 0.01. *** *p* < 0.001.

**Table 6:** Relationship between different types of differences in filial piety and life satisfaction.

Variables	Model 1	Model 2	Model 3	Model 4	Model 5 (Female)	Model 6 (Male)
Region	−0.07	−0.08 *	−0.07 *	−0.08 *	−0.05	−0.10 *
Gender (male = 1)	0.10	0.10	0.07	0.05		
Marital status (with a spouse = 1)		0.16 *	0.15 *	0.13 *	0.23 *	0.05
Number of sons		0.09 **	0.09 **	0.10 **	0.13 **	0.08 *
Number of daughters		0.02	0.02	0.03	0.08	−0.01
Education level (primary school = 1)			0.11	0.13	0.19	0.12
Log (annual income)			0.15 ***	0.10 **	0.10 *	0.10 **
Log (annual expenditure)			0.07	0.09 *	0.07	0.10 *
Physical condition				0.24 ***	0.25 ***	0.24 ***
Low expectation–low income (reference group)						
Low expectation–high income	0.49 ***	0.47 ***	0.40 ***	0.35 ***	0.20	0.39 ***
High expectation–low income	0.01	0.01	0.04	0.04	0.12	−0.02
High expectation–high income	0.29 ***	0.27 ***	0.23 **	0.21 **	0.27 *	0.16
R^2^	5.12%	6.44%	10.66%	19.17%	20.67%	19.13%
df	5	8	11	12	11	11

* *p* < 0.05. ** *p* < 0.01. *** *p* < 0.001.

**Table 7:** Each dimension of filial piety and life satisfaction.

Variables	Model 1	Model 2	Model 3	Model 4	Model 5 (Female)	Model 6 (Male)
**Dimensions of Filial Piety Expectation**						
Care expectation	−0.02	−0.01	−0.01	0.01	0.02	−0.01
Expectation of respect	0.14 **	0.15 **	0.15 **	0.12 *	0.10	0.13 *
Greeting expectation	−0.05	−0.05	−0.05	−0.07	−0.03	−0.09
Pleasure expectation	0.07	0.07	0.05	0.03	0.05	0.01
Expectation of obedience	−0.07	−0.08 *	−0.08 *	−0.04	0.03	−0.07
Financial expectation	−0.08 **	−0.09 **	−0.06 *	−0.04	−0.10 *	−0.01
**Dimensions of Filial Piety Income**						
Care income	0.02	0.02	0.02	−0.01	0.02	−0.02
Respect income	0.14 **	0.14 **	0.13 **	0.09	0.19 *	0.04
Greeting income	0.06	0.05	0.05	0.05	−0.08	0.12
Pleasure income	−0.06	−0.05	−0.05	−0.02	0.11	−0.10
Obedience income	0.03	0.02	0.03	0.04	0.08	0.02
Financial income	0.17 ***	0.16 ***	0.12 ***	0.11 ***	0.03	0.16 ***
**Dimensions of Differences in filial piety**						
Care difference	0.01	0.01	0.01	−0.01	−0.01	−0.01
Respect difference	−0.03	−0.03	−0.04	−0.03	0.01	−0.06
Greeting difference	0.05	0.05	0.05	0.06	−0.03	0.10 *
Pleasure difference	−0.04	−0.03	−0.03	−0.01	0.04	−0.03
Obedience difference	0.08 *	0.08 *	0.08 *	0.06	0.02	0.08 *
Financial difference	0.12 ***	0.12 ***	0.09 ***	0.07 **	0.08 *	0.07 **

* *p* < 0.05. ** *p* < 0.01. *** *p* < 0.001. Note: this has the same logic as the model of filial piety expectation and filial piety income affecting life satisfaction. Model 1 adds region and gender; based on Model 1, Model 2 adds marital status, number of sons and daughters; Model 3 is based on Model 2, which adds education level, annual income and annual expenditure; Model 4 adds self-rated health based on Model 3; based on Model 4, Model 5 and Model 6 analyze gender difference through regression.

## Appendix A

### Questionnaire about the New Dilemma of Pension Security among Rural Elderly Living Alone in Sichuan Province

Dear Sir/Madam:

As a research group from the school of public administration of Sichuan University, the purpose of this study is to learn about the reality of old-age security of rural elderly living alone in Sichuan province, in the background of overall planning of urban and rural areas. After that, we will aim to improve the life quality of rural elderly living alone. You can give your answers anonymously. Your information will be greatly valued and will be strictly confidential, so you needn’t have any concern. Thank you for your cooperation!

Please choose the most appropriate option and tick it, or fill in the blanks. You can ask the investigators for help if you have any problem.

No. ________________

Address: ________________

Mobile: _________________ Contact Person: ________________

**A. Basic Information**

A0 Interviewer record the Respondent’s address:

  1. Qionglai 2. Leshan 3. Suining

A1-1 Gender: 1. Male 2. Female

A1-2 What’s your date of birth? ____Year.

A1-3 What is your marital status?

  1. Married and spouse is alive

  2. Divorced

  3. Widowed

  4. Never married

A1-4 What is your level of education?

  1. No formal education

  2. Primary school and below

  3. Middle school and below

  4. High school, Vocational school, and below

  5. Associate degree and below

  6. Bachelor’s degree and above

A1-5 How many people are there in your household? _____.

   The number of sons_____. The number of daughters_____.

A1-6 How much cash did your children give you last year?

   _____yuan from sons. ____yuan from daughters.

A1-7 How much cash did you give your children last year?

   _____yuan to sons. ____yuan to daughters.

**B. Filial Piety Scale**

B1-6.1 How much do you expect to be cared for by your children?

   1. not at all 2. very little 3. on average 4. not a little 5. very much

B1-6.2 Whether your children actually provide you with care?

   1. not at all 2. very little 3. on average 4. not a little 5. very much

B1-6.3 How much do you expect to be respected by your children?

   1. not at all 2. very little 3. on average 4. not a little 5. very much

B1-6.4 Whether your children actually provide you with respect?

   1. not at all 2. very little 3. on average 4. not a little 5. very much

B1-6.5 How much do you expect to be greeted by your children?

   1. not at all 2. very little 3. on average 4. not a little 5. very much

B1-6.6 Whether your children actually provide you with greeting?

   1. not at all 2. very little 3. on average 4. not a little 5. very much

B1-6.7 How much do you expect to be pleased by your children?

   1. not at all 2. very little 3. on average 4. not a little 5. very much

B1-6.8 Whether your children actually provide you with pleasure?

   1. not at all 2. very little 3. on average 4. not a little 5. very much

B1-6.9 How much do you expect to be obeyed by your children?

   1. not at all 2. very little 3. on average 4. not a little 5. very much

B1-6.10 Whether your children actually provide you with obedience?

   1. not at all 2. very little 3. on average 4. not a little 5. very much

B1-6.11 How much do you expect to be financially supported by your children?

   1. not at all 2. very little 3. on average 4. not a little 5. very much

B1-6.12 Whether your children actually provide you with financial support?

   1. not at all 2. very little 3. on average 4. not a little 5. very much

**C. Other Related Issues**

C1-7 Where do you live now?

   1. Live alone in town 2. Live alone in rural area

   3. Live with family members in town 4. Live with family members in rural area

C1-7.1 With whom are you living together? (check all that apply)

  1. Spouse 2. Grandchildren (son’s children)

  3. Grandchildren (daughter’s children) 4. Other, please specify___

C1-8 Your average monthly income: _____yuan.

  annual income: ____yuan.

C1-9 What is your main financial resource? (check all that apply)

  1. Wage income: ___yuan per month

  2. Children’s financial support: ___yuan per month

  3. Minimum subsistence allowance: ____yuan per month

  4. Pension income: ____yuan per month

  5. Relatives’ financial support: _____yuan per month

  6. Farming income: ____yuan per month

  7. Other, please specify___

C1-9.1 What is the most one among above options? (check only one)

  1. Wage income

  2. Children’s financial support

  3. Minimum subsistence allowance

  4. Pension income

  5. Relatives’ financial support

  6. Farming income:

  7. Other, please specify___

C1-10 Your monthly expenditure: ____yuan. medical expenses: ___yuan.

  accommodation, transport, food and clothing expenses: ___yuan.

C1-11 I am satisfied with my life.

  1. Totally disagree 2. Disagree 3. Generally, agree 4. Agree

  5. Fully agree

C1-12 Who provide you with following supports?

    (Tick the corresponding box and check all that apply)

Family support	1. spouse	2. children	3. siblings	4. neighbors	The most important provider	No provider
Activities of daily living						
Financial support						
Emotional support						

C1-13 By whom do you want to be cared for?

  1. Son 2. Daughter 3. Son or daughter 4. Rely on yourself

C1-14 Have you ever provided your children with following supports?

	Yes	No
Support each other financially		
Support each other in food, clothing and household goods		
Support each other in housework, such as shopping, cooking, fixing things, doing cleaning, caring for children		
Comfort and encourage each other		

Do you have any other suggestion for old-age security of rural elderly living alone? __________________________

The questionnaire survey is finished, thank you for your support!
